# The Warm Phase of CRPS Type-1: Is It Time to Review the Budapest Criteria?

**DOI:** 10.3390/diagnostics15182397

**Published:** 2025-09-20

**Authors:** Gianantonio Saviola, Sergio Rosini, Luigi Molfetta, Luca Dalle Carbonare, Nazzarena Malavolta, Nunzia Di Meglio, Maria Antonietta Mazzei, Maurizio Muratore, Bruno Frediani

**Affiliations:** 1 Rheumatology Unit, Clinical Scientific Institutes Maugeri IRCCS, Castel Goffredo, 46042 Mantua, Italy; 2 Biomaterial Research Center, 57121 Livorno, Italy; 3 DISC Department, School of Medical and Pharmaceutical Sciences, Research Center of Osteoporosis and Osteoarticular Pathologies, University of Genoa, 16132 Genoa, Italy; 4 Department of Engineering for Innovation Medicine, University of Verona, 37129 Verona, Italy; 5 Casa di Cura Madre Fortunata Toniolo, 40141 Bologna, Italy; 6 Unit of Diagnostic Imaging, Department of Medical, Surgical and Neuro Sciences and of Radiological Sciences, University of Siena, Azienda Ospedaliero-Universitaria Senese, 53100 Siena, Italy; 7 Rheumatology Unit, Fazzi Hospital, 73100 Lecce, Italy; 8 Rheumatology Unit, University of Siena, Azienda Ospedaliero-Universitaria Senese, 53100 Siena, Italy

**Keywords:** CRPS type-1, complex regional pain syndrome, algodistrophy, bone marrow edema, bisphosphonates, neridronate, clodronate, hyperbaric oxygen treatment, pulsed electromagnetic fields

## Abstract

Complex Regional Pain Syndrome (CRPS) type 1 is a painful and disabling localized syndrome with a pathogenesis that is still unclear. The last revised diagnostic criteria for CRPS-1 syndrome were developed in 2012 (the so-called Budapest criteria), based only on clinical features, while the presence of bone marrow edema (BME) and the response to treatments were completely absent. As BME is usually present on magnetic resonance imaging (MRI) in the early (“warm”) phase of CRPS-1, this criterion should be added as a necessary criterion to Budapest criteria. In addition, hyperalgesia and/or allodynia are also commonly present in the warm phase. Therefore, both of these symptoms should be included as essential criteria. Furthermore, the response to bisphosphonates may be another important parameter to add to the list of treatment options, as well as hyperbaric oxygen therapy. Finally, it must be clear that BME is not an exclusive finding of CRPS-1. Therefore, a correct clinical history and, if needed, further radiological studies and laboratory tests should be performed to avoid a false diagnosis. In this paper, the “Bone Marrow Edema Diagnosis and Therapeutic Treatment” Italian Group (GEODEIT) proposes a revision of Budapest’s criteria to make them more meaningful and effective in reaching a correct and quick diagnosis of the disease.

## 1. Introduction

Complex Regional Pain Syndrome type 1 (CRPS-1) is a hyperalgesic syndrome first identified and described in 1900 by the surgeon Paul Herman Martin Sudeck, who published a paper on the subject in 1908 [1]. In this paper, Sudeck interpreted the pathology as inflammatory bone atrophy. He also described several features that are now considered characteristic of the disease. However, in the subsequent century, the condition now recognized as Complex Regional Pain Syndrome was referred to by a multitude of names, some of which proved to be misleading, inspired by either clinical or pathogenetic mechanisms. Indeed, the syndrome was not the exclusive domain of any single discipline, as specialists from diverse backgrounds competed for its recognition [2]. The unfortunate result was that diagnosis was almost invariably delayed, sometimes by years, while the patient had to endure painful and sometimes unbearable symptoms. In certain cases, the amputation of a portion of the affected limb was proposed and executed [3,4]. Indeed, the plethora of pharmacological (and non-pharmacological) treatments that have been proposed have largely proven ineffective, with the notable exception of hyperbaric oxygen therapy (HBOT), which has demonstrated a degree of success in several cases [5], and there are some reports on the usefulness of pulsed electromagnetic fields [6]. However, the advent of bisphosphonates has led to a paradigm shift in the management of bone marrow edema (BME), with substantial evidence supporting their efficacy in alleviating pain and inflammation. The advent of magnetic resonance imaging (MRI) as a radiological modality capable of clearly highlighting the condition has only recently enabled its identification. It was subsequently established that BME, present in numerous bone diseases, is also the radiological marker of CRPS-1, at least in the warm phase [7].

## 2. Applied Methodology

The Nominal Group Technique (NGT) was utilized to achieve consensus among experts regarding the proposed revisions to the diagnostic criteria for CRPS-1. The expert panel comprised nine members of the Italian Group for the Diagnosis and Therapeutic Treatment of Bone Marrow Edema (GEODEIT), including two orthopedic surgeons, two radiologists, one internist, and four rheumatologists. All members of the panel had extensive experience in musculoskeletal diseases and performed the in-person consensus method in a working session during the 7th annual congress of the society in 2024. The process was overseen by a trained facilitator, who ensured that the four key stages of the classic NGT protocol were adhered to, and that an environment of constructive collaboration was fostered [8]. This structured approach ensured the involvement of all members and facilitated the consideration of diverse perspectives [9,10]. Initially, each participant independently listed their key criteria and concerns about CRPS-1, followed by a round-robin sharing session to consolidate ideas without interruption. Following the consolidation of analogous items, the panel engaged in a detailed discussion to clarify and refine each point, thereby fostering mutual understanding. The session concluded with an anonymous prioritization process, with the combined rankings informing the final set of recommendations.

## 3. Bone Marrow Edema

**Figure 1:**
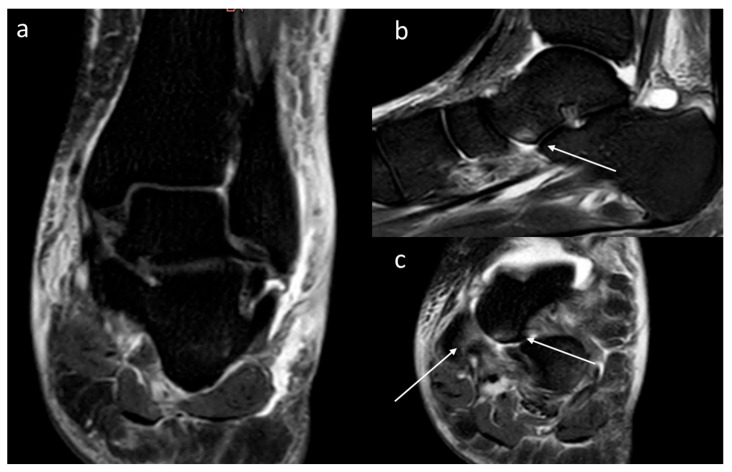
An example of bone marrow edema in an MRI examination in a patient with joint swelling, persistent pain, and allodynia on physical examination two months after a minor compressive trauma. The clinical diagnosis was CRPS in the warm phase. The MRI examination shows talo-tibial joint effusion, as well as significant thickening and diffuse edema of the subcutaneous tissue in the coronal Short Tau Inversion Recovery (STIR) sequence (**a**). Additionally, some focal and patchy areas of subchondral bone edema are visible at the level of the anterior portion of the talus in the sagittal (white arrows in (**b**)) and axial (**c**) STIR sequences, and at the level of the navicular bone (white arrow in (**c**)).

The concept of BME was originally introduced in 1988, in the early days of MRI, when some radiologists observed an image that they interpreted as an increase in the water content of the bone marrow, and termed “transient osteoporosis” [11]. The study identified 10 patients with advanced osteoarthritis of the hip and knee, with associated pain, in whom T1- and T2-weighted MRI sequences showed an increase in the fluid component of the subchondral bone marrow. In subsequent years, BME evolved from an occasional, difficult-to-interpret finding to a radiological marker of non-specific bone disease, which can occur in various clinical scenarios [7]. Within the domain of inflammatory diseases, it is prevalent in rheumatoid arthritis, psoriatic arthritis, spondyloarthritis, and gout, with the clinical significance of osteitis [12,13,14,15]. In the context of osteoarthritis, the presence of BME within the subchondral bone has been observed to be a consequence of RANKL-mediated inflammation, as well as the action of inflammatory cytokines, at least in those patients in whom osteoarthritis has an osteoporotic evolution. This phenomenon arises from repeated microtrauma, misalignment, obesity, or limb-length heterometry [16,17,18,19,20]. Furthermore, BME is invariably present in bone marrow edema syndromes (BMESs), pathologies characterized by hyperalgesia, with a generally favorable prognosis and an uncommon occurrence [21,22]. Conversely, CRPS-1, otherwise referred to as algodystrophy, manifests more frequently and is associated with a less favorable prognosis. MRI is typically utilized in cases where CRPS-1 is suspected to exclude differential diagnoses. Its diagnostic utility extends beyond the detection of soft tissue changes, such as contrast-enhanced thickening of periarticular, subcutaneous, and/or skin tissue, and joint effusion accompanied by synovial hypertrophy. Additionally, it serves to identify the presence of BME, which manifests with a diffuse or patchy, peripheral, subcortical, non-articular, and non-weight-bearing distribution (Figure 1) [23]. BME appears to be related to increased permeability of small intramedullary and soft tissue vessels due to not fully identified pathways, including hemodynamic abnormalities due to abnormal sympathetic function, and it may be present in up to 50% of cases [24]. In particular, the presence of BME is more frequent during the warm phase, with a tendency to fluctuate in intensity and migrate during the natural regression of the initial warm phase, and it is often absent during the cold phase of the disease: this is supported by histological studies showing a prevalence of pro-inflammatory mediators and increased bone resorption activity in the warm phase, with alterations that tend to diminish in the chronic phase [25]. The inability to consistently detect BME, which is often transient and easily overlooked, has resulted in its exclusion from the diagnostic criteria [26,27,28]. However, there are no recent studies in the literature evaluating the differential prevalence of BME in the warm and cold phases of CRPS-1, which is generally assessed without regard for the variability in the phase of disease activity [29].

## 4. CRPS-1: Clinical Features

The precise pathogenesis of CRPS-1 remains unclear; however, the available data suggest a central role for bone subjected to trauma or microtrauma, which manifests as an inflammatory response, at least in the initial phase [30]. It is also conceivable that hypovitaminosis D and osteoporosis may constitute risk factors [31]. The sequence of events that would lead to hyperalgesia and allodynia is a topic of ongoing debate, probably reflecting the involvement of the central nervous system (CNS). More recently, the role of ATP as a possible mediator of the syndrome has been highlighted [32]. CRPS-1 is a localized hyperalgesic syndrome, predominantly involving the peripheral joints of the foot, ankle, hand, and wrist, which do not coincide with the innervation territory of a single nerve branch or any dermatome. The manifestation of these symptoms is often characterized by a discrepancy between the quantitative and temporal dimensions, relative to the event that triggered them. The manifestation of these symptoms is characterized by the presence of hyperalgesia and/or allodynia, the latter being defined as pain induced by a stimulus or an event that is not typically algogenic [33]. The condition is most often observed as a complication of a fracture (particularly a wrist fracture), a sprain, or surgery [34,35,36,37]. However, it should be noted that there are many other possible triggers, including the use of barbiturates or isoniazid, tuberculosis, certain bone tumors, myocardial infarction, septic arthritis, polyneuropathies, cerebral vasculopathies, and psychosocial factors. In addition, 10% of cases are labelled as idiopathic [38,39,40,41]. The pathophysiology in the warm phase is dominated by persistent inflammation, with elevated pro-inflammatory mediators sensitizing peripheral and central nociceptive pathways, which underlie the development of allodynia. Studies have shown that allodynia and hyperalgesia are more prominent in the early, warm phase and may diminish as the disease transitions to the chronic, cold phase [42,43,44].

The syndrome generally manifests a few weeks after the triggering event, with an initial warm phase. In this phase, in addition to clear signs of inflammation, characterized by obvious swelling with translucent skin, other vasomotor, sudomotor/edematous, and motor/trophic symptoms are often present. In this phase, bone scintigraphy (for which Tc99-labeled bisphosphonate is used) is effective in detecting increased uptake and BME [24,45,46,47]. However, the appearance of ‘macular osteoporosis’ on traditional X-rays only becomes apparent after 1–2 months. The initial phase of the condition may subside spontaneously within a few months, or it may gradually shift to a ‘cold’ phase over a highly variable period of time in which the pain persists, generally less intensely, while many of the secondary symptoms disappear, giving way to cyanosis, hypothermia, joint stiffness, and subcutaneous hypotrophy. In this phase, bone scintigraphy no longer shows hyperuptake. Consequently, bisphosphonates, which tend to concentrate where there is high bone turnover due to their mechanism of action, may become ineffective [48,49]. The syndrome’s subsequent progression leads to atrophy with “claw hand” features, which has prompted extreme therapeutic interventions, such as surgical amputation. Finally, syndromes with a ‘cold’ onset and other phenotypes that are difficult to classify within the above classification are described [50].

## 5. CRPS-1: Diagnosis

As time has passed, a series of diagnostic criteria have been formulated to identify CRPS [51]. These criteria are based on the differences in symptoms found in patients with CRPS and in subjects with neuropathic pain without CRPS. Nevertheless, this method of assessment has been demonstrated to be inadequate for providing a precise diagnosis. Subsequently, based on further clinical experience and following additional diagnostic validation studies, the International Association for the Study of Pain (IASP) formulated a further set of guidelines known as the ‘Budapest criteria’ in 2012 (Table 1, [52]).

However, these diagnostic criteria do not allow for differentiation of the clinical characteristics of CRPS from skin damage secondary to trauma or from an uncomplicated fracture in the various stages of the disease. The prevailing conceptions surrounding CRPS posit the almost constant presence of BME in cases of definite CRPS, as opposed to the presence of skin edema solely as a consequence of ‘sudomotor’ or traumatic events. The aforementioned criteria encompass solely clinical signs and symptoms, whilst radiological criteria and responses to treatment are not factored into the assessment. In addition, allodynia and hyperalgesia are regarded as non-essential criteria for diagnosis. The GEODEIT group has therefore made the following observations:

- In the hyperalgesic syndrome of the warm phase, it is evident that pain, frequently manifesting as allodynia (a consequence of heightened sensitivity within the central nervous system), must be the primary diagnostic criterion.
- It is evident that BME should be regarded as the second fundamental criterion, given its value as a marker of CRPS-1.

It is not uncommon for MRI radiological reports, in the presence of BME, to reach overly hasty diagnostic conclusions: a comprehensive medical history and diagnosis must invariably be given due consideration.

In order to initiate the most appropriate treatment promptly, alternative diagnoses should be ruled out whenever possible. It is important to note that a delayed diagnosis of conditions such as rheumatoid arthritis can lead to irreversible damage due to the limited therapeutic window for this disease, which frequently leads to severe disability, lasting only a few months.

Consequently, it is our considered opinion that the response to bisphosphonates, in particular neridronate and clodronate, cannot fail to find a place within the diagnostic criteria for CRPS-1. It is important to note that neridronate has been approved for the treatment of CRPS-1 for a decade, while clodronate has been shown to be significantly effective since 2000 [53,54]. Bisphosphonates have demonstrated moderate-certainty evidence for pain reduction in CRPS, particularly in the early phase with bone edema, as shown in meta-analyses and randomized controlled trials [55,56,57]. The American Academy of Pain Medicine notes that bisphosphonates may be more effective in patients with imaging evidence of bone involvement (e.g., BME or osteopenia), but this is a therapeutic consideration, not a diagnostic criterion [39].

Similarly, HBOT has shown efficacy in symptom relief and functional improvement in CRPS, including in the warm phase, but its response is not used diagnostically [5,58,59].

Moreover, the use of bisphosphonates and HBOT represents a key approach to reducing pain symptoms, which is a fundamental prerequisite for enabling the application of rehabilitation strategies. Rehabilitation strategies for CRPS, such as graded motor imagery and mirror therapy, are effective non-invasive treatments, leading to significant improvements in both pain reduction and functional recovery [60].

Accordingly, the GEODEIT group thus proposes a revision of the criteria for the warm phase of CRPS-1, as indicated in Table 2.

## 6. Discussion

The most recent revised diagnostic criteria for CRPS-1 syndrome were developed in 2012 (referred to as the Budapest criteria) and were exclusively based on clinical factors. The presence of BME and the response to treatment were not taken into consideration. A number of studies have indicated that BME may be present in a variable proportion of patients with CRPS-1, yet it is absent in a significant percentage of cases. Moreover, the absence of BME does not preclude the diagnosis of CRPS-1; imaging is primarily utilized to rule out alternative diagnoses rather than to confirm CRPS-1 [26]. However, studies on the diagnostic role of MRI are dated and date back to the 1990s and 2000s, when MRI was still a limited method [26,61]. At present, MRI is a widely used and crucial method in the diagnostic and therapeutic management of osteoarticular diseases. Furthermore, improvements in technology have led to the development of sequences with adipose tissue signal suppression, which are highly sensitive to the presence of BME [62]. A 2020 study of 22 patients with CRPS-1 of the foot found that approximately 50% of subjects with a confirmed diagnosis had BME on MRI [24]. It should be noted that only five of the patients studied were in the warm phase of the disease. It is imperative to re-evaluate the role of MRI in the diagnosis of CRPS-1 by analyzing the patterns of BME present, particularly in the warm phase of the disease. Indeed, the extant studies are based on a limited number of cases and rarely consider the different imaging findings in relation to the phase of the disease (warm versus cold phase). It has been established that BME is present in a transient manner, particularly during the warm phase of CRPS-1, and its absence may be overlooked if MRI is conducted at a late stage [29].

The objective of GEODEIT is to establish a novel set of criteria for the early diagnosis of the CRPS-1 warm phase, with the aim of avoiding misdiagnosis and facilitating the administration of appropriate treatment in a timely manner.

As MRI frequently demonstrates the presence of BME in the initial (“warm”) phase of CRPS-1, we argue that this parameter should be incorporated into the diagnostic criteria. Furthermore, given that hyperalgesia and allodynia are invariably present during the warm phase of the disease, these symptoms must be incorporated into the diagnostic criteria, not as a secondary parameter but as a fundamental element of the diagnostic process. Nonetheless, the response to bisphosphonates and/or HBOT, if administered, could be incorporated as a secondary parameter to confirm the diagnosis. Rehabilitation strategies—including physical therapy, graded motor imagery, and mirror therapy—are recommended as first-line treatments for improving pain and disability in CRPS, with evidence supporting their efficacy for symptom management [60]; however, the therapeutic response to these interventions is not used diagnostically, and there is no evidence in the medical literature that a positive or negative response to rehabilitation should be considered in the diagnostic process for CRPS [39,63].

It could be argued that the criterion of response to bisphosphonates is misplaced. However, patients who present to a specialist (typically a rheumatologist, internist, or orthopedic surgeon) are aware of the reality of their condition. These patients have frequently been subjected to a plethora of ineffective therapeutic interventions. In some cases, they have been administered bisphosphonates, albeit with an inadequate dosage, yielding only marginal and transient benefits. In other instances, although the dosage of bisphosphonates was appropriate, these patients have subsequently experienced a recurrence of symptoms that appear to challenge the initial diagnosis. However, it is acknowledged that bisphosphonates have proven efficacy in the treatment of BME. Consequently, the therapeutic success achieved through their utilization cannot be considered the sole diagnostic parameter. Our group aims to pursue, as a forthcoming research project, the validation of the proposed diagnostic criteria by testing their effectiveness in clinical practice, in order to assess their applicability and potential impact on the management of CRPS.

## 7. Conclusions

It is this author’s opinion that the revised Budapest criteria are deficient in a fundamental aspect. The term “MRI” is employed, which is known almost invariably to reveal the presence of BME. It is evident that the proposed diagnostic revision primarily focuses on the initial, warm phase of CRPS-1, to avert erroneous diagnoses that result in delays. This finding potentially signifies a diagnostic limitation concerning the subsequent phases of the disease, namely the cold and atrophic phases. Nevertheless, a meticulous review of the patient’s medical history, in conjunction with the application of the Budapest criteria, would enable accurate clinical categorization.

It is essential to note that therapeutic interventions are significantly more effective during the warm phase of CRPS-1, underscoring the importance of timely and accurate diagnosis.

## Figures and Tables

**Table 1:** Budapest criteria [52].

(1) Continuing pain, which is disproportionate to any inciting event.
(2) Must report at least one symptom in three of the four following categories: - Sensory: reports of hyperesthesia and/or allodynia - Vasomotor: reports of temperature asymmetry and/or skin color changes and/or skin color asymmetry - Sudomotor/edema: reports of edema and/or sweating changes and/or sweating asymmetry - Motor/trophic: reports of decreased range of motion and/or motor dysfunction (weakness, tremor, dystonia) and/or trophic changes (hair, nail, skin)
(3) Must display at least one sign at the time of evaluation in two or more of the following categories: - Sensory: evidence of hyperalgesia (to pinprick) and/or allodynia (to light touch and/or deep somatic pressure and/or joint movement) - Vasomotor: evidence of temperature asymmetry and/or skin color changes and/or asymmetry - Sudomotor/edema: evidence of edema and/or sweating changes and/or sweating asymmetry - Motor/trophic: evidence of decreased range of motion and/or motor dysfunction (weakness, tremor, dystonia) and/or trophic changes (hair, nail, skin)
(4) There is no other diagnosis that better explains the signs and symptoms

**Table 2:** Revised diagnostic criteria for the warm phase of CRPS-1, proposed by GEODEIT in 2025.

Criteria to Be Displayed at the Time of Assessment:
(1) Evidence of continuing pain, disproportionate, with hyperalgesia (to pinprick) and/or allodynia (to light touch and/or deep somatic pressure and/or joint movement) ^a^
(2) Evident swelling in the hands or feet ^a^
(3) No other diagnosis better explains the signs and symptoms (i.e., osteoarthritis, bmes, and inflammatory chronic arthritis must be excluded) ^a^
(4) Can be present or reported ^b^ - BME on MRI - Absence of inflammatory indicators - Therapeutic response: to an appropriate cycle of neridronate, clodronate, or hyperbaric oxygen treatment - Vasomotor: evidence of temperature asymmetry and/or skin colour changes and/or skin color asymmetry - Sudomotor/Edema: evidence of edema and/or sweating changes and/or sweating asymmetry - Motor/Trophic: evidence of decreased range of motion and/or motor dysfunction (weakness, tremor, dystonia) and/or trophic changes (hair, nail, skin)
At least **three** criteria are required for the fulfilment of CRPS-1 diagnosis.

^a^ Most common findings. ^b^ Findings helpful in diagnosis.

## Data Availability

No new data were created or analyzed in this study. Data sharing is not applicable to this article.

## Abbreviations

The following abbreviations are used in this manuscript:

BME	Bone Marrow Edema
BMES	Bone Marrow Edema Syndrome
CPRS	Complex Pain Regional Syndrome
GEODEIT	Bone Marrow Edema Diagnosis and Therapeutic Treatment Italian Group
